# Impact of Water Conductivity on the Structure and Swelling Dynamics of E-Beam Cross-Linked Hydrogels

**DOI:** 10.3390/gels11080611

**Published:** 2025-08-04

**Authors:** Elena Mănăilă, Ion Călina, Anca Scărișoreanu, Maria Demeter, Gabriela Crăciun, Marius Dumitru

**Affiliations:** National Institute for Laser, Plasma and Radiation Physics, 409 Atomistilor St., 077125 Magurele, Romania; elena.manaila@inflpr.ro (E.M.); anca.scarisoreanu@inflpr.ro (A.S.); maria.dumitrascu@inflpr.ro (M.D.); gabriela.craciun@inflpr.ro (G.C.); marius.dumitru@inflpr.ro (M.D.)

**Keywords:** superabsorbent hydrogel, electron beam radiation, water electrical conductivity, kinetics, sodium alginate

## Abstract

Prolonged drought and soil degradation severely affect soil fertility and limit crop productivity. Superabsorbent hydrogels offer an effective solution for improving water retention in soil and supporting plant growth. In this work, we examined the performance of superabsorbent hydrogels based on sodium alginate, acrylic acid (AA), and poly (ethylene oxide) (PEO) cross-linked with 12.5 kGy using e-beam irradiation. The hydrogels were assessed in various aqueous environments by examining network characteristics, swelling capacity, and swelling kinetics to evaluate the impact of water’s electrical conductivity (which ranges from 0.05 to 321 μS/cm). Morphological and chemical structure changes were evaluated using SEM and FTIR techniques. The results demonstrated that water conductivity significantly affected the physicochemical properties of the hydrogels. Swelling behavior showed notable sensitivity to electrical conductivity variations, with swelling degrees reaching 28,400% at 5 μS/cm and 14,000% at 321 μS/cm, following first-order and second-order kinetics. FTIR analysis confirmed that structural modifications correlated with water conductivity, particularly affecting the O–H, C–H, and COOH groups sensitive to the ionic environment. SEM characterization revealed a porous morphology with an interconnected microporous network that facilitates efficient water diffusion. These hydrogels show exceptional swelling capacity and are promising candidates for sustainable agriculture applications.

## 1. Introduction

Superabsorbent hydrogels exhibit a remarkable capacity for water absorption and retention, enabling them to capture and hold water from the soil, thereby reducing evaporative losses and enhancing soil fertility by maintaining optimal moisture levels. This mechanism contributes to increased water use efficiency and reduces the frequency of irrigation [1,2]. Owing to their outstanding functional properties, hydrogels are widely employed in the agricultural sector [3,4].

In agriculture, hydrogels are regarded as innovative materials that have garnered significant interest and extensive research attention, offering critical technological support for enhancing agricultural productivity. Moreover, hydrogels can be utilized for the adsorption of heavy metals from soil [5,6], as well as for the controlled and sustained release of fertilizers and pesticides [7]. These features contribute to improved quality and efficiency in agricultural production, enabling the implementation of more precise and intelligent management strategies that support the sustainable development of agriculture. Several studies have investigated various hydrogel compositions with high water absorption capacity used as soil conditioners to reduce drying and cracking [8,9,10], as well as for the controlled release of pesticides [11,12].

Alginate represents a promising class of polymers for hydrogel applications in agriculture. This natural biopolymer is distinguished by a favorable property profile and is widely used in hydrogel formulations due to its sustainable, non-toxic, biodegradable, and environmentally friendly nature [13,14]. However, the intrinsic limitations of alginate, such as its low mechanical strength and limited thermal stability, have hindered its large-scale applicability [15]. To overcome these challenges, numerous studies have investigated the incorporation of synthetic polymers, such as polyacrylic acid (PAA). Synthetic hydrogels derived from acrylates are noted for their superior mechanical properties and high water absorption capacity [16,17].

Radiation treatment can promote enhanced cross-linking between natural and synthetic polymers by intensifying the formation of macroradicals, which subsequently lead to the development of a three-dimensional, cross-linked network [18]. Elbarbary et al. synthesized superabsorbent hydrogels based on sodium alginate/poly (acrylamide)/chitosan via gamma irradiation and demonstrated that the developed hydrogels had a significant impact on plant quality and crop yield, thereby confirming their potential as soil amendments [19]. Seeponkai et al. prepared a PVA/PAA-based hydrogel membrane, revealing remarkable water absorption and ionic permeability properties. The study highlighted that the use of this hydrogel membrane in hydroponic or soilless cultivation supports clean agriculture, eliminating the need for pesticides or other chemical agricultural agents [20].

Current studies on conductive hydrogels have focused almost exclusively on enhancing their conductivity through the incorporation of conductive materials, such as conductive polymers [21], salts, or carbon-based compounds [22,23]. The structure and properties of hydrogels are significantly influenced by the presence and type of ions in water, as these ions directly affect how water interacts with the polymer network, leading to changes in swelling behavior, water mobility, and the internal organization of the hydrogel matrix [24,25]. Water is a fundamental resource for agriculture, plant cultivation, and crop production. An effective strategy to reduce water consumption involves the use of materials with high water absorption and retention capacity [26].

Although hydrogels have been extensively investigated with respect to their physicochemical and swelling behavior, no previous studies have directly examined the impact of water conductivity on these characteristics [27]. Given that water serves as the continuous phase within the three-dimensional hydrogel network, and considering that dissolved ions can interact with the functional groups of polymer chains, it is reasonable to expect that the electrical conductivity of the aqueous environment significantly influences the structural and physicochemical behavior of hydrogels.

In this study, we aimed to investigate the influence of water conductivity on the physicochemical, structural, and morphological properties of hydrogels obtained through e-beam irradiation. A single hydrogel formulation and a cross-linking method (12.5 kGy) were employed. Subsequently, the samples were immersed in five types of water with conductivities ranging from 0.055 μS/cm to 321 μS/cm to evaluate the role of ionic content during post-synthesis swelling and characterization. The results provide novel insights into the effect of water conductivity on hydrogel behavior, offering valuable guidance for the development of superabsorbent materials tailored for agricultural applications. Specifically, these findings support the enhancement of soil water retention and the controlled release of nutrients through the use of water with optimized ionic content and electrical conductivity.

## 2. Results and Discussion

### 2.1. Network Parameters

The effect of water conductivity on the network structure of hydrogels produced through e-beam cross-linking at a dose of 12.5 kGy was examined. The study focused on several crucial structural parameters for assessing the degree of cross-linking and the physicochemical characteristics of the hydrogels: gel fraction (G), cross-link density (q), network pore size (ξ), and porosity (P).

**Figure 1:**
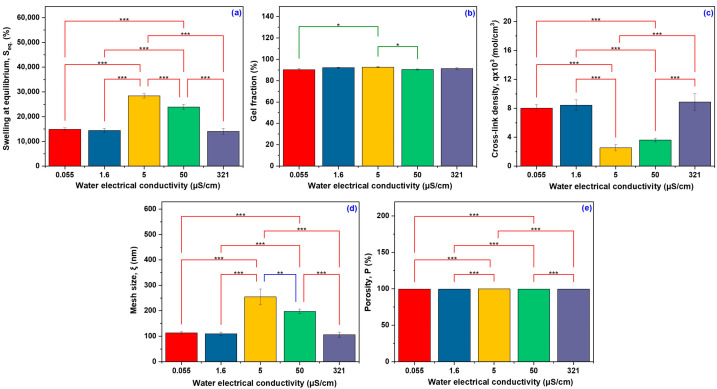
The significant difference between the water types used in swelling experiments and network parameters: swelling at equilibrium (**a**), gel fraction (**b**), cross-link density (**c**), mesh size (**d**), and porosity (**e**) (Fisher’s LSD Multiple Comparison Test; * significant at *p* < 0.05, ** significant at *p* < 0.01, and *** significant at *p* < 0.001.

The network characteristics were linked to the water conductivity to highlight how the ionic environment composition affects the behavior of the polymer network. Table 1 displays the obtained results describing the specific hydrogel network parameters. Figure 1 shows the degree of swelling and network parameters of hydrogels as a function of water conductivity. Fisher’s LSD Multiple Comparison Test was used to assess the comparison data and emphasize the findings of the experiments conducted on the hydrogel using the five different types of water with varying conductivities. Significant variations were observed across the different electrical conductivity conditions for most evaluated parameters. The S_eq_ (Figure 1a) exhibited pronounced differences (*p* < 0.001) between samples. An optimal ionic environment is important for maximizing the water retention capacity of the hydrogel, probably due to the balanced osmotic pressure and favorable ion–polymer interactions.

Based on the water’s conductivity, the experimental results of the gel fraction demonstrated very little variance.

Consequently, a gel fraction of 90.27 ± 1.06% was observed when 0.055 μS/cm deionized water was used, 92.11 ± 0.42% for distilled water with 1.6 μS/cm electrical conductivity, 92.66 ± 0.63% for demineralized water with 5 μS/cm, 90.40 ± 0.54% for the same category of water but with a higher conductivity of 50 μS/cm, and 91.26 ± 1.04% when tap water with an electrical conductivity of 321 μS/cm was used. These results suggest that variations in water conductivity do not significantly affect the gel fraction values, which remain high (above 90%) in all investigated hydrogels with only slight yet statistically significant differences *p* < 0.05 (Figure 1b). The slight variations observed may be attributed to differences in the ionic composition of water or the dissolution process of the soluble fraction during the extraction step. Nonetheless, the significant stability of the gel fraction shows a properly structured polymer network, featuring an increased level of cross-linking that provides hydrogels with stability in different water environments. Unlike the gel fraction, the water’s conductivity significantly influenced the cross-linking density.

Although there was no constant variation in the cross-linking density, it is clear that both very high and low electrical conductivity values result in the formation of a hydrogel with a strongly cross-linked network, in contrast to hydrogels hydrated in water with conductivities ranging from 5 to 50 μS/cm. In these conditions, the cross-linking density decreased up to 2.54 ± 0.42 mol/cm^3^, and at 50 μS/cm it slightly increased to 3.61 mol/cm^3^. The significant impact of water conductivity on cross-link density may be the result of a complex effect of the ions contained, which may act as partial inhibitors of polymer network formation reactions or affect the mobility of the chains [28].

The tap water (321 μS/cm) facilitated the formation of hydrogel with the highest cross-linking density up to a value of 8.87 ± 1.15 mol/cm^3^, and this could be attributed to the presence of specific salts (such as Ca^2+^ and Mg^2+^ ions) that may interact favorably with the hydrogel components, particularly with the carboxyl groups of AA and NaAlg. The q is inversely proportional to the equilibrium swelling of the hydrogel. The lowest cross-link density is observed at 5 µS/cm, corresponding to the highest swelling. Higher cross-link densities are found at conductivities where swelling is lower. A lower cross-link density leads to a looser network with a higher water absorption capacity [29]. In Figure 1c, the q showed significant variation (*p* < 0.001).

The formation of a polymer network with small mesh sizes, ranging from 112.88 ± 4.97 nm to 109.01 ± 6.77 nm, is favored by low water electrical conductivity values of 0.055 μS/cm and 1.6 μS/cm. A higher electrical conductivity of 321 μS/cm further helps in the reduction of network mesh sizes, reaching 105.63 ± 10.11 nm. This behavior is consistent with the theory of polymer networks, according to which a higher cross-linking density limits the mobility of polymer chains, restricts the swelling capacity (S_eq_ = 14,053 ± 8.16%), and leads to lower porosity (99.28 ± 0.06%).

In contrast, at the intermediate conductivities (5 μS/cm and 50 μS/cm), the polymer network presents a relaxed structure, characterized by significantly larger pore sizes (254.26 ± 31.58 nm and 196.89 ± 9.47 nm, respectively). The above values reflect more reduced cross-link density, which allows for a substantially higher swelling degree of 23,945 ± 4.12% and 28,428 ± 3.07%, respectively. As previously mentioned, water conductivity influences the cross-linking density and mesh size, but it is observed to have a lesser effect on porosity, which remains constant between 99.28 ± 0.06% and 99.66 ± 0.03%. The hydrogel structure’s sensitivity to the ionic composition of the solution is demonstrated by the change in mesh size and degree of swelling as a function of water conductivity (Figure 1d).

The largest mesh size is observed at 5 µS/cm (*p* < 0.001), indicating a more extensive network structure, which can retain a larger volume of water. Although variations in porosity were observed among the groups, as depicted in Figure 1e, these differences were not statistically significant (*p* > 0.001). This indicates that the hydrogels are highly porous, regardless of the ionic strength of the swelling medium. However, small variations suggest that ionic strength influences the polymer chains’ conformation and the pore network’s architecture. These changes do not significantly alter the total void volume but can have important consequences regarding the ease and speed with which water and other molecules can move through the hydrogel, altering its performance [28]. The changes in the ionic composition of the swelling medium have a substantial impact on the functional performance of hydrogels; therefore, these properties are essential for assessing hydrogels’ behavior.

Therefore, the results above indicate that the electrical conductivity of water can modify the swelling degree by modulating the electrostatic interactions between polymer chains, which in turn affect the rigidity and elasticity of the hydrogel. Moreover, the influence of conductivity is dependent on the cross-linking density of the polymer network, as a more densely cross-linked network tends to be less responsive to swelling changes induced by the ionic environment, thereby regulating the mechanical properties according to environmental conditions [30,31].

### 2.2. Swelling Degree of Hydrogels in Aqueous Media with Varying Electrical Conductivities

**Figure 2:**
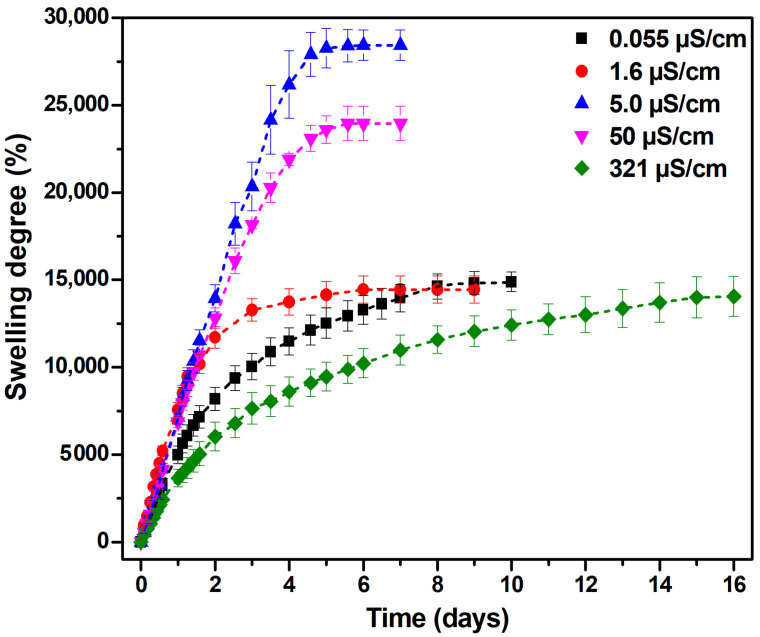
The effect of water’s electrical conductivity on hydrogel swelling.

The swelling capacity of superabsorbent hydrogels is significantly influenced by the physicochemical properties of the external medium, particularly its ionic strength and ionic valence. Both the electrostatic interactions between polymer chains and the osmotic pressure gradient across the hydrogel network are significantly affected by these characteristics [32]. For the first time, the swelling behavior of the superabsorbent hydrogel in water with different conductivities was investigated in this study. Figure 2 depicts the effect of water conductivity on hydrogel swelling.

The maximum swelling degree of the hydrogel, reaching 28,400%, was observed in demineralized water with an electrical conductivity of 5 μS/cm, suggesting that an intermediate ion content can facilitate hydrogel expansion through an optimal balance between charge shielding and osmotic pressure difference [33].

As electrical conductivity increases to 50 μS/cm, additional ions begin to shield the hydrogel’s hydrophilic charges, reducing its expansion capacity, and the swelling degree decreases to 24,000%. These results align with those of Trivedi et al. [34], who also demonstrated the maximal swelling performance of their hydrogel system (295.31 g/g or 29,531%) in low-conductivity water, thereby reinforcing the inverse relationship between ionic strength and swelling degree.

Additionally, hydrogels swollen in demineralized water reach equilibrium the fastest, in approximately 5 days. At very low conductivities (deionized water: 0.055 μS/cm; distilled water: 1.6 μS/cm), the swelling degree varies between 14,400% and 14,800%. The lowest swelling can be attributed to an insufficient concentration of ions to stabilize the hydrogel network, leading to potential structural collapse and thereby inhibiting further absorption.

The lowest swelling degree, approximately 14,000%, was recorded in tap water with a high electrical conductivity of 321 μS/cm, confirming the adverse effect of mineral-rich environments on the hydration of hydrogels. The elevated electrical conductivity of tap water reflects a considerable concentration of dissolved ionic species (i.e., Ca^2+^, Mg^2+^, Na^+^, Cl^−^), which compromise the osmotic driving force and electrostatic repulsion essential for swelling.

Moreover, polyvalent cations, such as Ca^2+^ and Mg^2+^, can form ionic cross-links with anionic sites on the polymer backbone, effectively tightening the hydrogel network and limiting its capacity to absorb water. Structurally, these interactions prevent water infiltration by causing the network to compress, decreasing the mesh size (105.63 nm, see Table 1), and decreasing the osmotic swelling pressure. When formulating hydrogels for agriculture, where ion concentration and water availability can differ significantly, the ionic environment must be carefully controlled [35].

In conclusion, the substantial difference of 14,400% between the maximum and minimum swelling values (i.e., 28,400% in demineralized water vs. 14,000% in tap water) underscores the critical role of water conductivity in determining hydrogel performance.

### 2.3. Swelling Kinetics vs. Water Electrical Conductivity

**Figure 3:**
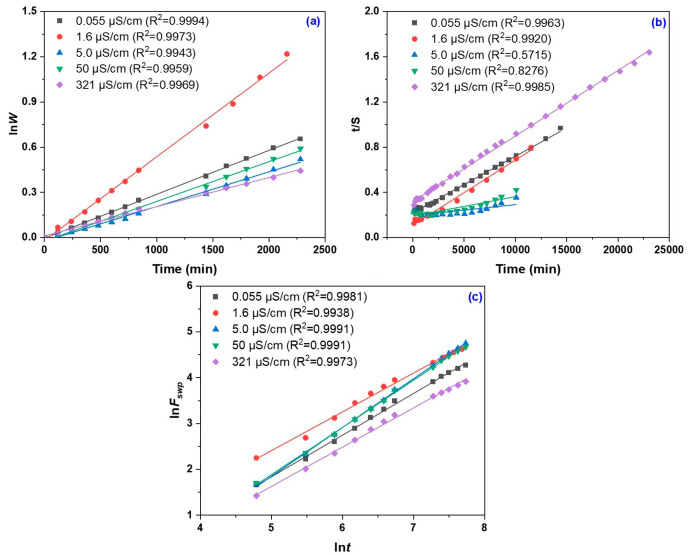
First-order swelling kinetics (**a**), second-order swelling kinetics (**b**), plots of lnF vs. *lnt* for the determination of n and k (**c**).

The plot of lnW vs. time (t) exhibits a linear trend (Figure 3a), indicating that a first-order kinetic model efficiently describes the swelling behavior of the hydrogel. The linear correlation implies that the water uptake process is controlled by a single rate-limiting step, most likely associated with the diffusion of water through the polymeric network. The rate constant k_1,S_ attains its maximum value at an electrical conductivity of 1.6 µS/cm, indicative of rapid swelling in distilled water (Table 1).

At very low conductivity (0.055 µS/cm), the swelling rate is moderate, whereas at high electrical conductivity (321 µS/cm), the process is markedly decelerated. This behavior can be attributed to the presence of salts, which shield the hydrophilic ionic groups within the hydrogel network, thereby reducing its capacity for rapid water absorption. Consequently, salts negatively impact the swelling kinetics by modifying electrostatic interactions and destabilizing the network’s structure.

Figure 3b presents the t/S versus t plot used to determine both the theoretical equilibrium swelling capacity (S_eq_) and the second-order kinetic constant (k_2,S_), with the corresponding values summarized in Table 2. The lower k_2,S_ values observed at conductivities of 5 and 50 µS/cm suggest a slow swelling process, likely due to internal ionic interactions that hinder water diffusion. In contrast, the increase in k_2,S_ at 321 µS/cm suggests a structural modification of the hydrogel network; although the initial swelling rate is lower, equilibrium is reached more rapidly. This behavior may indicate the formation of a denser network or a reorganization of polymer chains in response to higher salt concentrations.

**Figure 4:**
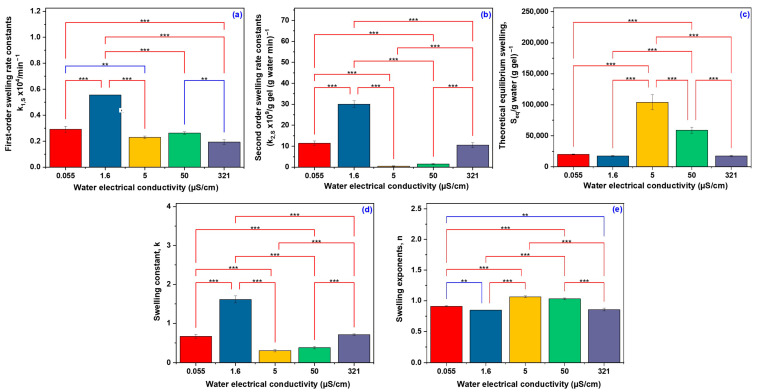
Significant difference between the water types used in the determination of swelling and water diffusion mechanisms: first-order swelling rate constants (**a**), second-order swelling rate constants (**b**), theoretical equilibrium swelling (**c**), swelling constants (**d**), and swelling exponents (**e**) (Fisher’s LSD Multiple Comparison Test; ** significant at *p* < 0.01, and *** significant at *p* < 0.001).

Figure 4 presents a statistical analysis of the swelling behavior and water diffusion mechanisms of hydrogels in water with different conductivities. The analysis was conducted using the LSD Fisher Multiple Comparison Test. As shown in Figure 4a,b, both the first-order swelling rate and the second-order swelling rate constants were significantly influenced by water conductivity (*p* < 0.001). The highest swelling rate was observed at a conductivity of 1.6 µS/cm, which was significantly greater than at 0.055 µS/cm and markedly higher than at 321 µS/cm. Notably, the maximum equilibrium swelling at 5 µS/cm indicates an optimal ionic environment that facilitates water uptake by the hydrogel.

The S_eq_ value at 5 µS/cm suggests a slow yet extensive swelling process, characteristic of a more open network structure that permits substantial water penetration at a reduced diffusion rate. In contrast, the lower swelling capacities observed at both high (321 µS/cm) and very low (0.055 µS/cm) conductivities point to a more compact network structure or the influence of osmotic effects that restrict water absorption.

The kinetic parameters k and n were determined based on the lnF vs. lnt plots illustrated in Figure 3c, with the corresponding values presented in Table 3. The values of the rate constant k indicate a rapid initial response of the hydrogel upon contact with water, with the highest value recorded at a conductivity of 1.6 µS/cm. These data suggest that salt-free distilled water enhances hydrophilic interactions and accelerates the swelling process and network equilibrium processes. Low k values correspond to a slower swelling process and a larger absorption capacity at conductivities of 5 and 50 µS/cm.

Figure 4c illustrates a distinct behavior of the S_eq_ at 5 µS/cm, which was significantly higher than under all other tested conditions (*p* < 0.001). This finding suggests that water with this specific ionic strength allows the hydrogel to swell to its maximum capacity compared to other conductivities. The lower S_eq_ values observed at both lower and higher conductivities indicate that the ionic environment influences the hydrogel’s ability to retain water at equilibrium.

A similar trend was observed for the overall swelling rate constant k in Figure 4d, with high statistical significance (*p* < 0.001). The parameter k reflects aspects of the initial and later stages of swelling. The observed trend follows the corresponding one for k_1_,_S_ and k_2_,_S_, having the highest value at 1.6 µS/cm, which means that the most favorable overall swelling kinetics occurs at this conductivity. In ultra-pure water (low conductivity), the concentration of ions present outside of the hydrogel is minimal. Although there is an initial driving force for water to enter the hydrogel due to the higher concentration of polymer, the lack of sufficient external ions could limit the full development of osmotic pressure. The hydrogel can swell, but not to its maximum capacity [36].

In the case of deionized and distilled water (0.055–1.6 µS/cm), the values of the exponent n, ranging between 0.847 and 0.909, indicate an anomalous (non-Fickian) transport mechanism, characterized by a combined contribution of water diffusion and polymer network relaxation. This behavior reflects a complex interaction between the water molecules and the rearrangement of the polymer matrix. The *n* value presented in Figure 4e shows several significant differences between groups. At conductivities of 5 and 50 µS/cm, *n* values greater than 1 indicate a super-diffusive regime dominated by network relaxation, typical of a flexible system that swells slowly but extensively under the influence of ionic interactions. For tap water (321 µS/cm), the *n* value of 0.855 suggests a return to an anomalous transport regime with a mixed mechanism, clearly affected by the presence of ions. In comparison to extreme conductivity conditions, these intermediate values highlight the enhanced ability of polymer chains to reorganize, exerting greater control over water uptake kinetics. This suggests that the ionic strength significantly modulates the dynamic behavior of the polymer network during the swelling process, influencing the balance between water diffusion and polymer relaxation mechanisms [37].

The data illustrated in Figure 4 robustly demonstrate that statistical significance is consistently observed, with the majority of pairwise group comparisons yielding *p*-values less than 0.001. These results underscore the strong influence of water conductivity on the swelling behavior of the hydrogels, affecting both kinetic and equilibrium swelling parameters. Specific conductivities, particularly 1.6 and 5 µS/cm, were found to enhance the swelling kinetics, while extremely low or high values led to diminished swelling performance. This behavior is attributed to the impact of ionic strength on osmotic pressure and polymer network interactions.

### 2.4. FTIR Spectral Analysis of Hydrogels Treated with Water of Varying Electrical Conductivity

**Figure 5:**
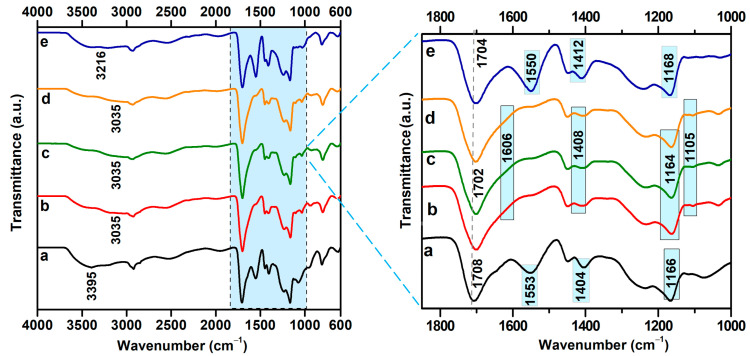
ATR-FTIR spectra of hydrogels immersed in water with varying electrical conductivities: (a) 0.055 µS/cm; (b) 1.6 µS/cm; (c) 5 µS/cm; (d) 50 µS/cm; (e) 321 µS/cm.

The ATR-FTIR spectra of the irradiated hydrogels immersed in water with different conductivities are presented in Figure 5. The hydrogel irradiated at a dose of 12.5 kGy exhibited distinct characteristic absorption bands at 3041 cm^−1^ (νO–H), 2947 cm^−1^ (νC–H), 1698 cm^−1^ (νC=O), 1550 cm^−1^ (νC=O), 1449 cm^−1^ (δCH_2_), 1412 cm^−1^ (δCOO^−^), 1235 cm^−1^ (νC–O), and 1164 cm^−1^ (νC–O–C) [38].

Immersion of the hydrogels in water with varying conductivity led to significant changes in their chemical structure, as evidenced by shifts in the characteristic absorption bands. The broad band associated with O–H stretching vibrations shifted within the range of 3035–3212 cm^−1^ with increasing water conductivity, suggesting a reorganization of the hydrogen bonding network within the hydrogel matrix, which is dependent on the ionic composition of the immersion medium. This pronounced variability in the O–H band position indicates a high sensitivity of hydrogen-bonding interactions to the ionic strength of the surrounding environment.

Slight shifts of the carbonyl band from 1698 cm^−1^ to 1702 cm^−1^ were observed for samples immersed in water with conductivities of 1.6, 5, and 50 μS/cm and to 1708 cm^−1^ for the sample in 0.055 μS/cm water. These shifts indicate alterations in the hydrogen-bonding environment of carboxylic acid (COOH) groups, suggesting possible changes in local polarity or the degree of ionization. The band at 1550 cm^−1^, characteristic of sodium alginate, shifted to higher wavenumbers (1606 cm^−1^) for samples exposed to water with conductivities of 1.6, 5, and 50 μS/cm, indicating the formation of carboxylate salts through ionic interactions with cations present in the aqueous medium. At the highest conductivity analyzed (321 μS/cm), this band reverted to 1550 cm^−1^, which may suggest a charge screening effect or a restoration of the initial coordination state of the COO^−^ groups due to the high ion concentration [39].

Regardless of the conductivity conditions, the band at 1449 cm^−1^ remained unchanged, indicating the presence of moderate ionic interactions between water cations and carboxylate groups. Additionally, the band at 1412 cm^−1^ exhibited only minor shifts, appearing at 1404 cm^−1^ for 0.055 μS/cm and 1407 cm^−1^ for 50 μS/cm conductivity.

Significant changes were observed in the spectral region corresponding to C–O–C groups. In water with the lowest conductivity (0.055 μS/cm), the band at 1164 cm^−1^ shifted to 1075 cm^−1^, at intermediate conductivities, it stabilized around 1105 cm^−1^, and at the highest conductivity, it was detected at 1118 cm^−1^. These shifts indicate that interactions between water ions and the ether groups of PEO influence the hydrogen bonding network within the hydrogel matrix.

The stretching vibration band of the C–H bonds, located at 2947 cm^−1^ and present in all three analyzed polymers, exhibited a shift toward lower frequencies following immersion in various types of water. The positions of the ν(C–H) band and the Δν values corresponding to the average stretching vibration frequency of C–H before and after immersion in water with different conductivities are presented in Table 4.

Notably, the magnitude of the frequency shift of this band correlates with the cross-link density data. Specifically, hydrogels immersed in water with conductivities of 0.055 µS/cm and 1.6 µS/cm exhibited more pronounced shifts (Δν = 26 cm^−1^ and Δν = 23 cm^−1^, respectively) compared to the hydrogel immersed in water with a conductivity of 5 µS/cm, which showed a smaller shift (Δν = 14 cm^−1^), and this observation is consistent with the lower cross-link density.

In the case of the C–O–C group, a significant shift in the average frequency was observed in the FTIR spectra for all investigated conductivity values. However, the most pronounced shift was recorded for the sample with a conductivity of 0.055 µS/cm, where the frequency shift reached Δν = 85 cm^−1^, suggesting stronger molecular interactions or structural reorganization at this conductivity level.

ATR-FTIR spectral analysis confirms the direct influence of the ionic composition of the immersion medium on the structure and reactivity of the irradiated hydrogels. The most significant spectral changes occur in the regions associated with the asymmetric stretching vibrations of the COO^−^ and O–H groups, underscoring the high sensitivity of these functional groups to the ionic environment.

Water conductivity affects the chemical structure of the hydrogel in a differentiated manner, likely through alterations in the degree of ionization of functional groups and the reorganization of the hydrogel’s three-dimensional network.

### 2.5. SEM Morphological Analysis of Hydrogels Treated with Water of Varying Electrical Conductivity

**Figure 6:**
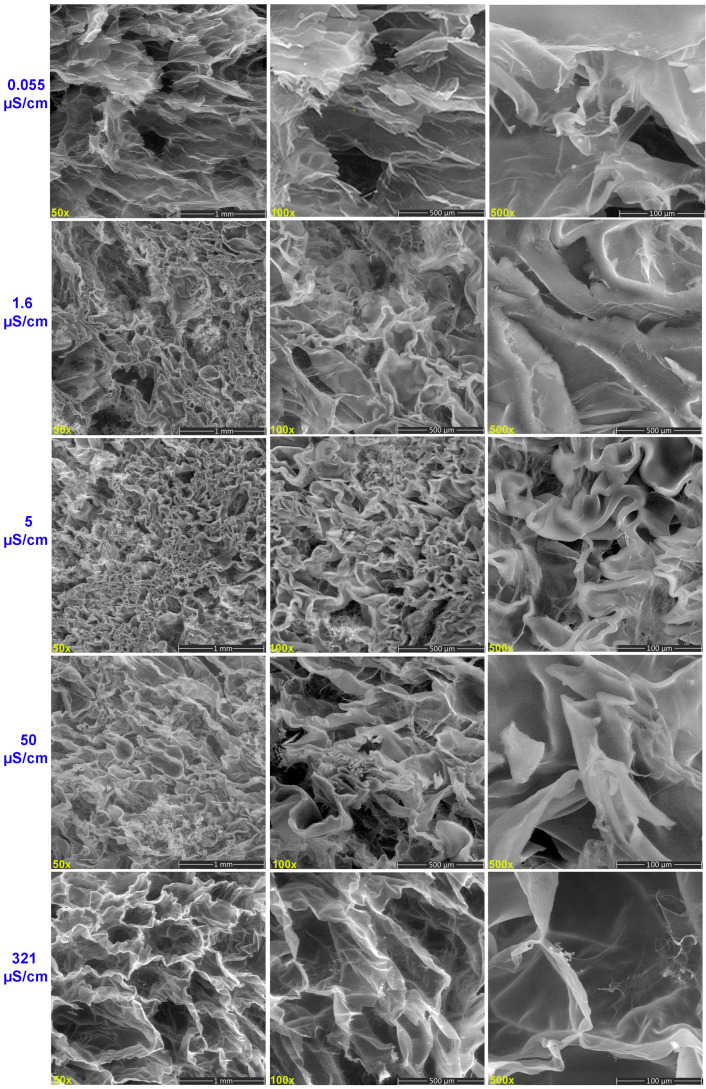
SEM images of hydrogels exposed to water with different electrical conductivities.

SEM images reveal that the hydrogels have an irregular and non-porous structure when they are swollen in water with very low conductivity (Figure 6). At 0.055 μS/cm, lower magnifications reveal a hydrogel with a flake-like structure with very thin walls, along with a few randomly distributed small holes. When the hydrogel was hydrated with water of 1.6 μS/cm, its structure started to present morphologically distinct areas, probably due to the formation of water absorption channels. At high magnifications, a hydrogel with a slightly shrunken continuous structure is observed. Based on this finding, the results show a strong correlation with the swelling study, which yielded a mild swelling ratio and increased cross-link density.

SEM images of the cross-section of the hydrogels swollen with water of 5 and 50 µS/cm conductivity exhibited well-defined, interconnected, three-dimensional porous structures featuring significant micropores, suggesting a hydrogel structure containing water permeation regions into which water easily diffused.

In aqueous environments with a conductivity of 5 µS/cm, the reduced ionic strength significantly limits the formation of ionic cross-links within the hydrogel network. This is due to the reduced concentration of cations capable of electrostatic interactions with anionic sites on the polymer chains. As a result, the hydrogel network architecture becomes less densely cross-linked and more loosely organized, as further substantiated by the FTIR analysis, which indicates structural alterations consistent with diminished cross-linking efficiency. The diminished complexation between available cations and functional groups—primarily carboxylate and hydroxyl moieties—on the hydrogel matrix leads to a more expanded network configuration.

Consequently, the hydrogel exhibits increased swelling capacity. A higher swelling ratio is also caused by the electrostatic repulsion between the carboxyl and hydroxyl anionic groups, which expands the space between the polymer chains, increasing the pore size and facilitating the diffusion or easier diffusion of water molecules into the hydrogel. When hydrogels are hydrated with tap water (321 μS/cm), their network is porous, with pores of varying diameters and thin walls surrounding them. Its swelling tendency is sustained for up to 16 days when the hydrogel is hydrated with tap water, even though it absorbs less water. The stability of the hydrogel network is preserved for a longer amount of time by absorbing less water, which may make it easier to provide a prolonged moist environment. As the ionic strength rises, the degree of ionization of the carboxyl groups decreases, increasing the formation of hydrogen bonds. In low electrolyte solutions, ionized carboxylate groups electrostatically repel each other. As the electrolyte concentration increases, cations can shield the electrostatic repulsion. Based on these two mechanisms, a denser hydrogel network can be developed and prevent the further growth of swelling capacity [40].

## 3. Conclusions

Hydrogels based on sodium alginate, acrylic acid, and PEO cross-linked via e-beam irradiation demonstrated remarkable performance under varying water electrical conductivity conditions, highlighting the adaptive behavior of the polymer network in response to the ionic composition of the surrounding medium.

Water conductivity significantly influences the cross-linking density and mesh size of the polymer network, without any influence on gel fraction and porosity. At extreme conductivity values (very low (0.055 µS/cm) or very high (321 µS/cm)), the network becomes compacted, forming a dense structure with small pores and reduced swelling capacity. In contrast, intermediate conductivity levels favor more relaxed network architectures with larger mesh sizes and enhanced absorption, reflecting an optimal balance between network flexibility and ionic interactions.

The swelling capacity of the hydrogel is strongly influenced by water conductivity, reaching a maximum of 28,400% in demineralized water and decreasing significantly to 14,000% in highly conductive tap water. This outcome emphasizes the adverse impact of ions, particularly of polyvalent cations, on osmotic pressure and the polymer network structure, reinforcing the need to manage the ionic environment in practical applications, such as agriculture. The swelling kinetics predominantly follow first- and second-order mechanisms, with the diffusion rate governed by interactions between the polymer network and water, modulated by the ionic strength. Distilled water (1.6 µS/cm) enables rapid and efficient swelling, while tap water (321 µS/cm) slows both the swelling rate and capacity, reflecting a denser network.

The SEM morphological changes are consistent with swelling behavior and are further sustained by FTIR spectral data, which indicate that functional groups within the hydrogel structure undergo modifications influenced by water conductivity. These findings suggest that a more enlarged, less organized network emerges from weaker ionic interactions at lower ionic strengths, leading to decreased cross-link density. As a result of electrostatic repulsion and decreased complexation between functional groups and cations, the hydrogels show improved swelling capacity and pore size. The development of a porous structure with various pore diameters, further confirmed by hydration with tap water, highlights the enhanced role of ionic interactions.

Therefore, the careful selection of water type is crucial, as it directly impacts the absorption properties of the hydrogel, enhancing its performance in applications like water resource management, soil conditioning, and agriculture.

## 4. Materials and Methods

### 4.1. Materials

Sodium alginate (M_w_ = 120,000.00–190,000 g/mol), PEO (M_w_ = 300,000 g/mol), AA (M_w_ = 71.08 g/mol), and potassium persulfate (PP, M_w_ = 270.322 g/mol) were purchased from the Redox Group Company, Bucharest, Romania, and Sigma-Aldrich, Saint Louis, Missouri, US.

For the experimental investigations, five types of water with different conductivities were used:

(a) Deionized water (conductivity: 0.055 μS/cm; pH = 6.7) produced in the laboratory using TKA Pacific UP/UPW6 (Thermo Fisher Scientific, Niederelbert, Germany).
(b) Distilled water (conductivity: 1.6 μS/cm; pH = 6.05; ionic concentrations: Ca^2+^ < 0.09 mg/L, Na^+^ < 1 mg/L, Fe^3+^ < 0.01 mg/L, SO_4_^2−^ < 1 mg/L, NO_2_^−^ < 0.02 mg/L, NO_3_^−^ < 1 mg/L), also obtained in the laboratory using a GFL 2304 glass water still (LAUDA DR. R. WOBSER GMBH & CO. KG, Laudaplatz 1, Germany).
(c) Commercially demineralized water with medium-grade purity (conductivity: 5 μS/cm; pH = 5.9; ionic concentrations: Na^+^ < 0.01 mg/L, Fe^3+^ < 0.03 mg/L, CaCO_3_^−^ < 0.02 mg/L, SiO_2_ ≤ 1 mg/L), procured from Laborex S.R.L., Prahova, Romania.
(d) Commercially demineralized water (conductivity: 50 μS/cm; pH = 7; ionic concentrations: Na^+^ < 0.05 mg/L, Cl^–^ = 0.248 mg/L, Fe^3+^ < 0.03 mg/L, CaCO_3_^−^ < 0.05 mg/L, SiO_2_ ≤ 1 mg/L), procured from Laborex S.R.L., Prahova, Romania.
(e) Tap water (conductivity: 321 μS/cm; pH = 7.66; ionic concentrations: NH_4_^+^ < 0.05 mg/L, Ca^2+^ = 58.44 mg/L, Mg^2+^ = 35.44 mg/L, NO_2_^−^ < 0.033 mg/L, NO_3_^−^ = 5.02 mg/L), from the municipal distribution network of Măgurele, Romania.

### 4.2. E-Beam Radiation Synthesis of Hydrogels

The polymeric blend was prepared according to the previously described formulation [41], using 2% sodium alginate, 20% AA, 0.1% PEO, and 0.1% PP, all dissolved in 100 mL of distilled water. The reactants were then mixed thoroughly to ensure complete homogenization.

After homogenization, the mixture was transferred into medical-grade syringes with an internal diameter of 15 mm and irradiated using the 5.5 MeV linear accelerator (ALID 7) at the Electron Accelerator Laboratory of the National Institute of Laser, Plasma, and Radiation Physics, Bucharest, Romania. Irradiation was performed with an electron beam at a dose of 12.5 kGy and a dose rate of 0.9 kGy/min under atmospheric conditions at room temperature (25 °C). Radiation dosimetry was carried out with a graphite calorimeter, which serves as the primary standard for electron beam dosimetry [42].

**Figure 7:**
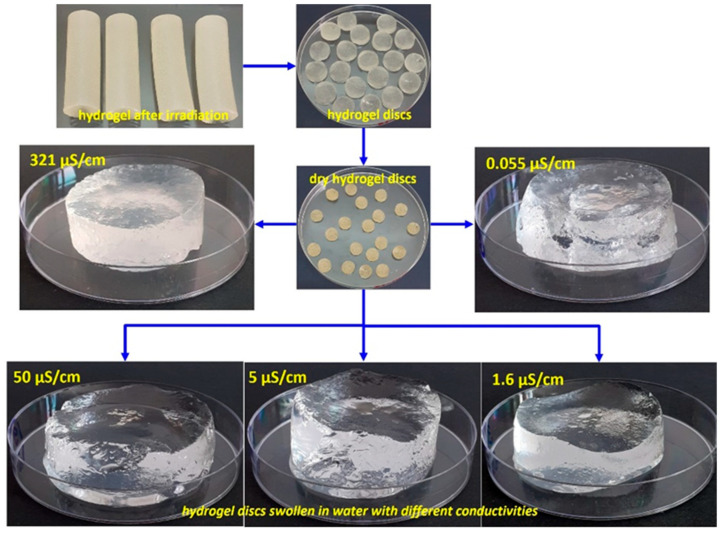
Representative digital images of hydrogels after irradiation, drying, and immersion in water with varying conductivity.

The irradiated hydrogels were stabilized at ambient temperature for 24 h. Subsequently, they were carefully extracted from the syringes, cut into 3–4 mm thick discs, and purified by washing them with ethanol to remove any residual unreacted monomers. The resulting samples were initially air-dried for 72 h, then placed in an oven at 50 °C for 24 h until they reached a stable weight, and finally stored in desiccators to maintain their anhydrous state. Digital images of the hydrogels after irradiation, drying, and immersion in water with varying conductivity are shown in Figure 7.

### 4.3. Analysis of Network Parameters

Following oven-drying at 50 °C, the initial weight of cross-linked hydrogels (*m*_0_) was measured. The samples were then immersed in the five types of water with varying conductivity at room temperature for 48 h to remove any soluble components, subsequently dried again at 50 °C, and weighed (*m_d_*). The gel fraction (G%) was calculated gravimetrically using Equation (1) [43].

(1) $$G\left(\%\right)=\frac{m_{d}}{m_{0}}\times 100$$

To further characterize the cross-linked polymer network, several structural parameters were evaluated, including the cross-linking density (q), mesh size (ξ), and porosity (P). Equations (2)–(6), used for the determination of these parameters, are well-established in the scientific literature [44,45,46].

(2) $$q=\frac{M_{c}}{M_{r}},$$

(3) $$M_{r}=\frac{\left(m_{NaAlg}\times M_{NaAlg}\right)+\left(m_{AA}\times M_{AA}\right)+\left(m_{PEO}\times M_{PEO}\right)}{m_{NaAlg}+m_{AA}+m_{PEO}},$$

(4) $$M_{C}=-V_{1}d_{P}\frac{\nu_{S}^{\frac{1}{3}}-\frac{\nu_{S}}{2}}{\ln(1-\nu_{S})+\nu_{S}+\chi \nu_{S}^{2}},$$

(5) $$\xi =\upsilon_{s}^{-1/3}\cdot l\sqrt{\frac{2C_{n}M_{c}}{M_{r}}},$$

(6) $$P(\%)=\frac{V_{d}}{1-V_{d}}\times 100,$$

where V_1_ represents the molar volume of the swelling agent (distilled water, 18 cm^3^/mol); d_p_ denotes the density of the hydrogel; υ_s_ (cm^3^) signifies the polymer volume fraction in the swollen state; l corresponds to the length of the C–C bond (0.154 nm); C_n_ is the Flory characteristic ratio; and V*_d_* denotes the volume ratio of water at equilibrium.

### 4.4. Swelling Behavior and Kinetics of the Hydrogels

Hydrogels with known dry weight (m_d_) and a diameter of 9.349 ± 0.014 mm were subjected to swelling over a period of 16 days. At predetermined time intervals, the samples were removed from the swelling medium, gently blotted with filter paper to eliminate excess surface moisture, and immediately weighed (m*_s_*). Additionally, the swelling behavior was evaluated in five aqueous media with electrical conductivities of 0.055, 1.6, 5, 50, and 321 µS/cm in order to investigate the effect of water conductivity on the hydrogel’s swelling capacity. The swelling degree (S%) was calculated according to Equation (7) [47].

(7) $$S\left(\%\right)=\frac{m_{s}-m_{d}}{m_{d}}\times 100$$

To investigate the swelling mechanism of hydrogels, several kinetic models were applied, including first- and second-order models, which enable the determination of swelling rate constants and the equilibrium swelling degree of (*S_eq_*) [48]. In the first-order analysis, the rate constant (k_1,S_)and the equilibrium swelling degree are calculated, whereas the second-order model (k_2,_*_S_*) additionally determines the initial swelling rate (r_0_) [49].

(8) dSdt=k1,S(Seq−S)

(9) $$lnW=k_{1,S}t\;$$

(10) dSdt=k2,S(Seq−S)2

(11) tS=A+Bt 

where W represents the swelling ratio, defined as W = S_eq_/(S_eq_ – S), *A* denotes the reciprocal of the initial swelling rate, expressed as either r_0_ or 1/k_2,S_ × S_eq_^2^, and *B* corresponds to the inverse of the equilibrium swelling degree, given by *B* = (1/S_eq_).

To assess the water diffusion mechanism within hydrogels, the swelling exponent (*n*) was calculated, indicating the type of water transport based on the diffusion behavior relative to polymer chain mobility. In this context, the values of *n* determine whether the diffusion follows Fickian (n ≈ 0.5), anomalous (0.5 < n < 1), or non-Fickian (n = 1 or n > 1) behavior [50,51].

(12) $$F_{swp}=\frac{M_{t}-M_{0}}{M_{0}}={kt}^{n}$$

(13) $$lnF_{swp}=nlnt+lnk\;$$

where *F_swp_* denotes the fractional swelling ratio at time t, M_t_ represents the weight of the swollen hydrogel at time t, and M_0_ corresponds to the weight of the dry hydrogel.

### 4.5. Evaluation of Chemical Structure and Morphological Features of the Hydrogel

The chemical structure and surface morphology of the hydrogels were evaluated using FTIR and SEM analyses. For these investigations, hydrogel samples were freeze-dried using a Biobase BK-FD18P lyophilizer (freezing at −85 °C and drying for 48 h).

Structural changes were analyzed using a Perkin Elmer Spectrum 100 spectrometer (Perkin Elmer, Waltham, MA, USA) equipped with a diamond crystal and operated in ATR mode. The spectral resolution was set at 4 cm^−1^, and each spectrum was averaged over 50 scans in the range of 4000–600 cm^−1^.

Surface morphology was examined using a scanning electron microscope (SEM), model FEI Inspect S, operating at 20 kV (FEI Co. Ltd., Hillsboro, OR, USA). Cross-sections of the freeze-dried hydrogels were analyzed after being coated with a thin layer of gold. The SEM images were acquired at magnifications of 50×, 100×, and 500×.

### 4.6. Statistical Analysis

Experimental data are expressed as mean ± standard deviation (SD) from three independent measurements. Statistical analysis was performed using one-way analysis of variance (ANOVA), followed by Fisher’s Least Significant Difference (LSD) post hoc test. A significance level of *p* < 0.05 was considered statistically relevant. All computations were carried out using Microsoft Excel 2021 and OriginPro V2023.

## Figures and Tables

**Table 1:** Physicochemical properties of hydrogels: equilibrium swelling (S_eq,_ %), gel fraction (G, %), cross-link density (q, mol/cm^3^), mesh size (ξ, nm), and porosity (P, %).

Water Electrical Conductivity (µS/cm)	S_eq_ (%)	G (%)	q ×10^3^ (mol/cm^3^)	ξ (nm)	P (%)
**0.055**	14,878 ± 3.77 ^c^	90.27 ± 1.06 ^b^	8.01 ± 0.51 ^a^	112.88 ± 4.97 ^c^	99.33 ± 0.03 ^b^
**1.6**	14,440 ± 5.33 ^c^	92.11 ± 0.42 ^ab^	8.43 ± 0.75 ^a^	109.01 ± 6.77 ^c^	99.31 ± 0.04 ^b^
**5**	28,428 ± 3.07 ^a^	92.66 ± 0.63 ^a^	2.54 ± 0.42 ^b^	254.26 ± 3.50 ^a^	99.66 ± 0.03 ^a^
**50**	23,945 ± 4.12 ^b^	90.40 ± 0.54 ^b^	3.61 ± 0.26 ^b^	196.89 ± 9.47 ^b^	99.58 ± 0.02 ^a^
**321**	14,053 ± 8.16 ^c^	91.26 ± 1.04 ^ab^	8.87 ± 1.15 ^a^	105.63 ± 10.11 ^c^	99.28 ± 0.06 ^b^
*F* value	166.1844	5.3225	55.8578	53.5606	69.4507
*p* value	<0.0001	0.01468	<0.0001	<0.0001	<0.0001

Significant differences are indicated by different lowercase superscripts for each dataset’s values within each column (*p* ≤ 0.05). Test results of one-way ANOVA: *F*-statistic (*F*-value) and *p*-value. If *p* < 0.05, the samples are significantly different. For the *F*-value, the number of groups (*k*) = 5, the number of samples (*n*) = 15, the degrees of freedom in the numerator (*df*_1_) = 4, and the degrees of freedom in the denominator (*df*_2_) = 10. The *F*-critical value is 3.4780.

**Table 2:** Results for swelling kinetics: first-order swelling rate constants (k_1,*S*_ ×10^4^/min^−1^), second-order swelling rate constants (k_2,*S*_ × 10^9^/g gel (g water min)^−1^), and theoretical equilibrium swelling (S_eq_/g water (g gel)^−1^).

Water Electrical Conductivity (µS/cm)	k_1,S_ ×10^4^/min^−1^	k_2,S_ ×10^9^/g gel	S_eq_ (Theoretical)/g Water
**0.055**	0.292 ± 0.023 ^b^	11.37 ± 1.07 ^b^	20,004 ± 639 ^c^
**1.6**	0.556 ± 0.001 ^a^	30.03 ± 1.59 ^a^	17,370 ± 919 ^c^
**5**	0.230 ± 0.011 ^cd^	0.49 ± 0.15 ^c^	104,087 ± 12,330 ^a^
**50**	0.263 ± 0.010 ^bc^	1.53 ± 0.26 ^c^	58,860 ± 4759 ^b^
**321**	0.193 ± 0.010 ^d^	10.54 ± 1.19 ^b^	17,259 ± 1182 ^c^
*F* value	285.5630	406.6245	123.2881
*p* value	<0.0001	<0.0001	<0.0001

The values represent the averages of three samples. Values within each column for each dataset with different lowercase superscripts are significantly different (*p* ≤ 0.05). Test results of one-way ANOVA: *F*-statistic (*F*-value) and *p*-value. If *p* < 0.05, the sample means are significantly different; if *p* > 0.05, the sample means are not significantly different. For the F-value, the number of groups (*k*) = 5, the number of samples (*n*) = 15, degrees of freedom in the numerator (*df*_1_) = 4, and degrees of freedom in the denominator (*df*_2_) = 10. The *F*-critical value is 3.4780.

**Table 3:** Results for swelling exponents (n) and swelling constants (k × 10).

Water Electrical Conductivity (µS/cm)	k × 10	n
**0.055**	0.666 ± 0.051 ^b^	0.909 ± 0.010 ^b^
**1.6**	1.612 ± 0.091 ^a^	0.847 ± 0.003 ^c^
**5**	0.309 ± 0.033 ^c^	1.065 ± 0.014 ^a^
**50**	0.379 ± 0.027 ^c^	1.031 ± 0.015 ^a^
**321**	0.706 ± 0.023 ^b^	0.855 ± 0.023 ^c^
*F* value	305.5099	145.50916
*p* value	<0.0001	<0.0001

The values represent the averages of three samples. Values within each column for each dataset with different lowercase superscripts are significantly different (*p* ≤ 0.05). Test results of one-way ANOVA: *F*-statistic (*F*-value) and *p*-value. If *p* < 0.05, the sample means are significantly different; if *p* > 0.05, the sample means are not significantly different. For the F-value, the number of groups (*k*) = 5, the number of samples (*n*) = 15, degrees of freedom in the numerator (*df*_1_) = 4, and degrees of freedom in the denominator (*df*_2_) = 10. The *F*-critical value is 3.4780.

**Table 4:** Positions of the ν(C–H) and ν(C–O–C) bands, along with Δν values, in water with different electrical conductivities.

Bands (cm^−1^)	Hydr. 12.5 kGy	Conductivity (µS/cm)						Δν (cm^−1^)			
		0.055	1.6	5	50	321	0.055	1.6	5	50	321
**ν(C–H)**	2947	2921	2924	2933	2929	2931	26	23	14	18	16
**ν(C–O–C)**	1164	1075	1105	1105	1105	1118	85	59	59	59	46

## Data Availability

The original contributions presented in this study are included in the article. Further inquiries can be directed to the corresponding author.
